# Cerebellar Purkinje cells incorporate immunoglobulins and immunotoxins in vitro: implications for human neurological disease and immunotherapeutics

**DOI:** 10.1186/1742-2094-6-31

**Published:** 2009-10-29

**Authors:** Kenneth E Hill, Susan A Clawson, John W Rose, Noel G Carlson, John E Greenlee

**Affiliations:** 1 Department of Neurology, University of Utah School of Medicine, 50 North Medical Drive Salt Lake City, UT 84132, USA; 2 Neurology Service, George E. Wahlen Veterans Affairs Medical Center, 500 Foothill Drive, Salt Lake City, UT 84148, USA; 3 Brain Institute, University of Utah, 383 Colorow Drive, Salt Lake City UT 84108, USA; 4 GRECC, George E. Wahlen Veterans Affairs Medical Center, 500 Foothill Drive, Salt Lake City, UT 84148, USA; 5 Department of Neurobiology and Anatomy, University of Utah School of Medicine, 50 North Medical Drive Salt Lake City, UT 84132, USA; 6 Center on Aging, University of Utah, 10 South 2000 East, Salt Lake City, UT 84112-5880, USA

## Abstract

**Background:**

Immunoglobulin G (IgG) antibodies reactive with intracellular neuronal proteins have been described in paraneoplastic and other autoimmune disorders. Because neurons have been thought impermeable to immunoglobulins, however, such antibodies have been considered unable to enter neurons and bind to their specific antigens during life. Cerebellar Purkinje cells - an important target in paraneoplastic and other autoimmune diseases - have been shown in experimental animals to incorporate a number of molecules from cerebrospinal fluid. IgG has also been detected in Purkinje cells studied post mortem. Despite the possible significance of these findings for human disease, immunoglobulin uptake by Purkinje cells has not been demonstrated in living tissue or studied systematically.

**Methods:**

To assess Purkinje cell uptake of immunoglobulins, organotypic cultures of rat cerebellum incubated with rat IgGs, human IgG, fluorescein-conjugated IgG, and rat IgM were studied by confocal microscopy in real time and following fixation. An IgG-daunorubicin immunotoxin was used to determine whether conjugation of pharmacological agents to IgG could be used to achieve Purkinje cell-specific drug delivery.

**Results:**

IgG uptake was detected in Purkinje cell processes after 4 hours of incubation and in Purkinje cell cytoplasm and nuclei by 24-48 hours. Uptake could be followed in real time using IgG-fluorochrome conjugates. Purkinje cells also incorporated IgM. Intracellular immunoglobulin did not affect Purkinje cell viability, and Purkinje cells cleared intracellular IgG or IgM within 24-48 hours after transfer to media lacking immunoglobulins. The IgG-daunomycin immunotoxin was also rapidly incorporated into Purkinje cells and caused extensive, cell-specific death within 8 hours. Purkinje cell death was not produced by unconjugated daunorubicin or control IgG.

**Conclusion:**

Purkinje cells in rat organotypic cultures incorporate and clear host (rat) and non-host (human or donkey) IgG or IgM, independent of the immunoglobulin's reactivity with Purkinje cell antigens. This property permits real-time study of immunoglobulin-Purkinje cell interaction using fluorochrome IgG conjugates, and can allow Purkinje cell-specific delivery of IgG-conjugated pharmacological agents. Antibodies to intracellular Purkinje cell proteins could potentially be incorporated intracellularly to produce cell injury. Antibodies used therapeutically, including immunotoxins, may also be taken up and cause Purkinje cell injury, even if they do not recognize Purkinje cell antigens.

## Background

Antibodies to cytoplasmic components of cerebellar Purkinje cells have been repeatedly described in sera and cerebrospinal fluid (CSF) of patients developing paraneoplastic cerebellar degeneration in the setting of systemic cancer, as well as in systemic lupus erythematosus and certain other disorders [1-7]. Despite their frequent detection, however, the roles of such antibodies in the pathogenesis of neuronal injury have been uncertain. Intact neurons have been thought to be essentially impermeable to IgG, and antibodies to cytoplasmic or nuclear neuronal antigens have been considered unable to enter neurons and bind to their intracellular target antigens during life [8].

A possible exception to neuronal exclusion of antibodies - and an important target in autoimmune neuronal injury - is the cerebellar Purkinje cell. In experimental animals, Purkinje cells have been shown capable of taking up a variety of substances from ventricular CSF, including propidium iodide, granular blue, bisbenzimide, and horseradish peroxidase conjugated to wheat germ agglutinin [9-11]. That Purkinje cells might also incorporate IgG has been suggested by several older studies using fixed cerebellar tissue. Fabian and Ritchie, in 1986, reported immunohistochemical staining for IgG in Purkinje cells and occasional other neurons in rat brains [12]. In subsequent studies, Karpiak detected antibodies to S100 protein within Purkinje cells following intraventricular injection [13]. Graus et al. detected both normal and anti-Yo IgG in Purkinje cells of guinea pigs sacrificed after intraventricular IgG instillation [14]; and our own work demonstrated IgG in Purkinje cells of rats sacrificed after intraperitoneal IgG injection of anti-Yo IgG in the presence of blood-brain barrier disruption [15]. In humans, uptake of IgG has been suggested by the detection of kappa and lambda light chains in Purkinje and certain other neurons of a patient dying of multiple myeloma [16]. Despite these observations, however, the ability of viable Purkinje cells to incorporate antibody has never been studied systematically, and the use of post mortem material in all published studies does not exclude the possibility of entry of IgG into neurons after death. The ability of Purkinje and related neurons to take up antibody is important not only because of the possible role of autoantibodies in disease causation but also because cerebellar injury and Purkinje cell destruction have been described in animals and human patients receiving IgG-conjugated immunotoxins [17-20].

Organotypic (slice) cultures of rat or mouse cerebellum provide a tissue culture system which allows sequential study of Purkinje cell interaction with exogenous compounds, including immunoglobulins, in a setting in which Purkinje and other neurons maintain normal anatomical relationships. In the present study, we demonstrate that cerebellar Purkinje cells are able to take up and clear both IgG and IgM during life. We also demonstrate that IgG uptake is not limited to immunoglobulins of the host species, that fluorochrome-conjugated IgGs can be used to follow IgG uptake in real time, and that targeted Purkinje cell destruction can be achieved following uptake of an immunotoxin whose IgG is not reactive with Purkinje cell antigens.

## Methods

### Immunoglobulins and immunoglobulin fluorochrome conjugates

Purified normal rat IgG, fluorescein-conjugated IgGs, and purified rat IgM were obtained commercially (Invitrogen, Carlsbad, CA). For immunotoxin studies and controls, the monoclonal rat IgG antibody GK1.5 (American Type Culture Collection; ATCC TIB-207), specific for mouse CD4+ cells was isolated from supernatant using fast protein liquid chromatography (FPLC)(Pharmacia, Nutley, New Jersey) [21]. Integrity of each IgG and IgM reagent was confirmed by subjecting each antibody to sodium dodecylsulfate polyacrylimide gel electrophoresis (SDS-PAGE) prior to its use in tissue culture experiments. To exclude the possibility that immunoglobulin fragments might develop during incubation experiments, media containing each immunoglobulin was analyzed by SDS-PAGE after being maintained for 24 hours at 37°C. Potential reactivity of the immunoglobulins for Purkinje cells was excluded by reacting each IgG and IgM employed in these studies with fixed and unfixed frozen sections of rat cerebellum.

### Preparation of daunorubicin-IgG immunotoxin

Daunorubicin (daunomycin) is an anthracycline antineoplastic agent which causes cytotoxicity by intercalating with cellular DNA to produce DNA double-strand breaks and by enhancing catalysis of oxidation-reduction reactions and generating oxygen free radicals [22]. The compound produces an intrinsic red fluorescence with an excitation of 488 nm and emission of 595 nm [23]. Work by Shen and Ryser demonstrated that conjugation of daunorubicin using a pH-sensitive cis-aconityl spacer resulted in a compound which was stable at neutral pH but released unaltered free daunorubicin at pH 4 or below [24]. Diener et al. subsequently demonstrated that coupling of daunorubicin by this method to a monoclonal antibody specific for T cells produced a conjugate which selectively eliminated the response of T lymphocytes to concanavalin A in vitro [25].

To determine whether uptake of daunorubicin conjugated to IgG could be used to target Purkinje cells specifically, daunorubicin was covalently linked to the purified rat monoclonal IgG, GK1.5, specific for mouse CD4+ cells [24]. GK1.5 was first shown not to react with rat cerebellar Purkinje cells in frozen or fixed section in either unconjugated or immunotoxin-conjugated forms (data not shown). Preparation of the immunotoxin employed the method of Shen et al. [24]. In brief, daunorubicin (Sigma-Aldrich, St. Louis MO) was dissolved in 200 mM Tris HCl pH 9.0 and the cis-aconitic anhydride (CAA)(Sigma) was dissolved in absolute ethanol. Daunomycin and the CAA spacer were mixed and reacted together at pH 9.0 for 15 minutes. The pH was then slowly lowered to 3.0 with 0.1N hydrochloric acid, and the mixture was incubated for 15 minutes at room temperature. The sample was centrifuged, and the pellet was resuspended in distilled water and adjusted to pH 8.0 using 0.1N sodium hydroxide. The daunomycin-CAA spacer mixture was then added to 1-ethyl-3-methyl carbodiimide (Sigma) and purified IgG at a mixture of 0.2:1.0:1.0, reacted overnight at room temperature, and dialyzed extensively against phosphate-buffered saline (PBS). The immunotoxin was quantified by using extinction coefficients for IgG and for daunorubicin, and integrity of the immunotoxin was established by SDS-PAGE (see Results). Efficacy of the immunotoxin had previously been proven by demonstrating its ability to produce killing within one hour of incubation in a lymphoproliferative stimulation assay using con-A activated mouse lymph node cells (data not shown) [25].

### Organotypic cerebellar cultures

**Figure 1:**
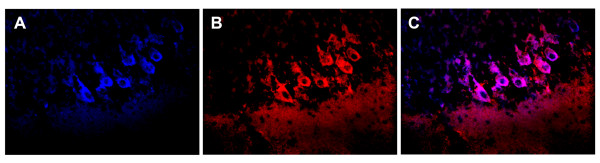
**Neurons incorporating IgG contain calbindin, identifying them as Purkinje cells**. Cultures were incubated with normal human IgG for intervals of up to 96 hours, fixed, permeabilized, and immunolabeled for calbindin (Panel A: blue) as a marker for Purkinje cells and IgG (Panel B: red). Panel C shows the merged image for both calbindin and IgG (magenta). Uptake of IgG is confined to cells expressing calbindin. (Objective magnification 40×)

All aspects of animal handling and care were conducted with local Institutional Animal Care and Use Committee (IACUC) approval in an Association for Assessment and Accreditation of Laboratory Animal Care (AAALAC)-approved facility (The Veterans Affairs Salt Lake City Health Care System Veterinary Medical Unit). The method used to prepare cultures was modified from that of Rothstein et al. [26]. In brief, 10-12 or 23-27 day old Sprague-Dawley rat pups (Charles River, Germantown, MD) were euthanized. Cerebellums were rapidly harvested and sectioned (200 μm) using a Lancer Vibratome, Series 1000 (Technical Products International, St. Louis, MO). Slices were collected in iced sterile Hank's balanced salt solution (Invitrogen) containing glucose (6.4 mg/ml) plus gentamicin (20 μg/ml). The cerebellar slices were transferred onto the surfaces of 0.4 μm pore size 30-mm Millipore Millicell-CM porous membranes (Millipore, Temecula, CA)(2 slices/membrane) in culture wells containing 1 ml of incubation medium with 25% heat inactivated horse serum, 25% Hanks balanced salt solution with 25.6 mg/ml glucose, and 50% minimum essential media (MEM) (Gibco/Invitrogen) with 25 mM of HEPES (Sigma), at a final pH of 7.2. Cultures were incubated at 37°C in a 5% CO_2_/95% humidified air environment overnight before being used for experiments and were then maintained with twice weekly changes of medium. Resultant cultures exhibited typical cerebellar morphology, with Purkinje cells clearly identifiable by morphology and by positive immunostaining of Purkinje cells in fixed cultures with both anti-Yo antibodies (data not shown) and antisera to recombinant calbindin (Millipore), a protein widely used as a marker for cerebellar Purkinje cells[27,28](Figure 1). Cultures for each time point were prepared from cerebellar slices from 4-6 animals. With the exception of real-time antibody uptake studies, which employed day 24 animals, all experiments were carried out using cultures derived from both younger (day 10-12) and older (day 23-27) animals. All studies were done in duplicate or triplicate. IgG concentrations were adjusted to 7.5 μg/ml for all experiments; we had found that this immunoglobulin concentration provided optimal imaging of antibody uptake with minimal background fluorescence. No difference was noted in survival of Purkinje or other neurons in cultures derived from younger as opposed to older animals.

### Studies of IgG uptake in living, unfixed cultures

The ability of living Purkinje cells to incorporate IgG in real time was studied by incubating cultures from 24 day old rats in media containing a 1:800 dilution of Cy5- or FITC-conjugated donkey anti-human IgG, using this age group because it more accurately reflects adult cerebellar structure. These antibodies had been previously shown not to label Purkinje cells in frozen section. Culture plates were maintained in a heated microscope stage incubator (SmartSlide microincubation chamber, WaferGen Biosystems, Fremont CA) and studied at intervals for up to 48 hours.

### Studies of IgG uptake and clearance using fixed cultures

To determine whether observations made in real time could be duplicated in fixed cultures and to assess the effect of intracellular IgG on cell survival, parallel cultures were incubated with 7.5 μg/ml concentrations of normal rat IgG. These cultures were fixed for study at intervals up to 120 hours. To determine whether Purkinje cells were able to clear incorporated IgG, organotypic cerebellar cultures were incubated with 7.5 μg/ml dilutions of normal rat IgG for 24 or 48 hours. Cultures were then placed in tissue culture media lacking rat IgG, and harvested at sequential intervals for 48 hours before being fixed, permeabilized, and labeled with Cy5-conjugated goat anti-rat IgG (Jackson ImmunoResearch Laboratories, Inc., West Grove, PA).

### Incubation with IgM

To determine whether Purkinje cells were able to incorporate larger immunoglobulin molecules, organotypic cultures were incubated in media containing 12.5 μg/ml of purified rat IgM. Cultures were harvested at serial intervals through 120 hours, fixed, permeabilized, and labeled with Cy5-conjugated goat anti-rat IgM (Jackson). Ability of Purkinje cells to clear IgM was assessed as described above for IgG.

### Incubation of cultures with Daunorubicin-IgG immunotoxin

Organotypic cerebellar cultures were incubated with 5 μg/ml of daunorubicin-IgG/ml and were examined after 4, 6, 8, 24, and 48 hours. Controls included cultures incubated for periods of up to 120 hours with unconjugated daunorubicin at a concentration equal to that present in the daunorubicin-IgG conjugate (500 nM) or with an equivalent concentration of the unconjugated monoclonal IgG.

### Determination of Purkinje cell viability and quantitation of Purkinje cell death

Purkinje cells in animals have been shown to incorporate a number of compounds from ventricular CSF including propidium iodide and ethidium homodimer which are excluded from most living cells and whose entry into cells has been used as a markers for cell death [29](Greenlee et al. submitted for publication). SYTOX green is a dye which, like propidium iodide and ethidium homodimer, is excluded from living cells but which readily enters dead or dying cells to bind to intracellular nucleic acids [29]. We have found that SYTOX green is excluded from viable Purkinje cells for periods of up to 24 hours but readily enters Purkinje cells following cell death induced by excitotoxic agents, detergents, or exposure to reduced temperature (Greenlee et al., submitted for publication). To quantify Purkinje cell death, cultures were incubated with either IgG or immunotoxin conjugates and SYTOX green (25 nM) was added two hour prior to fixation and immunofluorescence confocal microscopy. The amount of cell death was quantified by a blinded observer and consisted of counting of the number of cells labeled by Cy5 conjugated IgG containing or lacking SYTOX green. Live cells were recorded as containing Cy5-conjugated IgG and lacking SYTOX green. Dead cells were scored as cells co-labeled for IgG and SYTOX green. A minimum of four fields was examined for each time point and the percent of dead cells was calculated. Approximately 40-90 cells were counted for each field and the average percentage cell death was obtained from four fields captured at 40× magnification. Statistical significance between groups was examined using the non-parametric Mann-Whitney U-statistical analysis using GraphPad Instat statistical software (GraphPad Software, Inc., La Jolla, Ca).

### Immunofluorescence methods

Frozen sections of rat cerebellum used to exclude cross-reactivity of our antibodies with Purkinje cells were studied by indirect immunofluorescence using Cy5-conjugated goat anti-rat IgG (Jackson) as previously described [30,31]. Unfixed cultures studied for uptake of fluorochrome-conjugated IgG in real time used Cy-5 conjugated or FITC-conjugated donkey anti-human IgG antibodies, maintaining cultures in the microscope stage incubating chamber. Cultures for more detailed study of Cy5 or FITC-conjugated IgGs were fixed in 2% paraformaldehyde for 1 hour at room temperature but not permeabilized. To detect unconjugated IgG or IgM within Purkinje cells, cultures were fixed in 2% paraformaldehyde for 1 hour, permeabilized with 0.2% Triton X-100 for 1 hour 30 minutes, incubated overnight at 4°C with 1:800 dilutions of Cy5-conjugated donkey anti-human IgG or IgM, (Jackson), and then washed and cover slipped using Prolong Gold (Invitrogen).

### Identification of Purkinje cells

Cerebellar Purkinje cells are usually recognizable in histological sections or cultures by virtue of their distinctive morphology. In addition, this cell population can be identified by immunolabeling for calbindin. To confirm that cells incorporating immunoglobulins were Purkinje cells, cultures incubated with immunoglobulin G and were harvested at intervals through 96 hours. SYTOX green was added to cultures two hours before harvesting to assess cell viability. Cultures were then fixed in 2% paraformaldehyde, permeabilized with Triton X-100 as described above, and incubated for overnight at 4°C with a 1:1000 dilution of rabbit anti-calbindin antibody (Chemicon/Millipore AB1778, Temecula, CA). Cultures were then incubated overnight at 4°C with Cy5-conjugated donkey anti-human IgG (1:800) and with Cy3-conjugated donkey anti-rabbit IgG (1:800)(Jackson) to visualize calbindin, washed, and studied using confocal microscopy.

### Confocal microscopy

Confocal image acquisition employed a Nikon Eclipse E800 upright microscope (Nikon Biosciences, Melville, NY) and the Personal Confocal Microscopy PCM-2000 utilizing Argon-ion and green and red HeNe lasers to acquire images. Simple Personal Confocal Image software program (Compix, Cranberry Township, PA) was used for acquisition of digital images and image analysis. A red HeNe laser with a 633 nm excitation filter and 675 LP filter was used to visualize Cy5. A green HeNe laser with 543 excitation filter and 556 LP filter were used to visualize Cy3. The Argon-ion laser was used with a 488 filter for FITC, a 510 LP filter to image SYTOX green, and a 605 LP filter to visualize daunorubicin. All filters were matched to the peak emission spectrum of the fluorochromes employed. General procedures utilized individual fluorochromes with X, Y, and Z scans of 14-20 focal planes. Identical focal plane settings for each fluorochrome were used for single visual field analysis to ensure that each corresponding fluorochrome was imaged in the same focal plane. In all studies, stringent uniform experimental parameters and computer software settings were maintained for the respective image analyses. Because the vibratome preparation techniques used to prepare organotypic cultures invariably resulted in death of neurons on the cut surfaces of culture slices, image analysis in all experiments was confined to the interior portions of the cultures.

## Results

### Confirmation of IgG and IgM integrity and lack of reactivity with Purkinje cells

**Figure 2:**
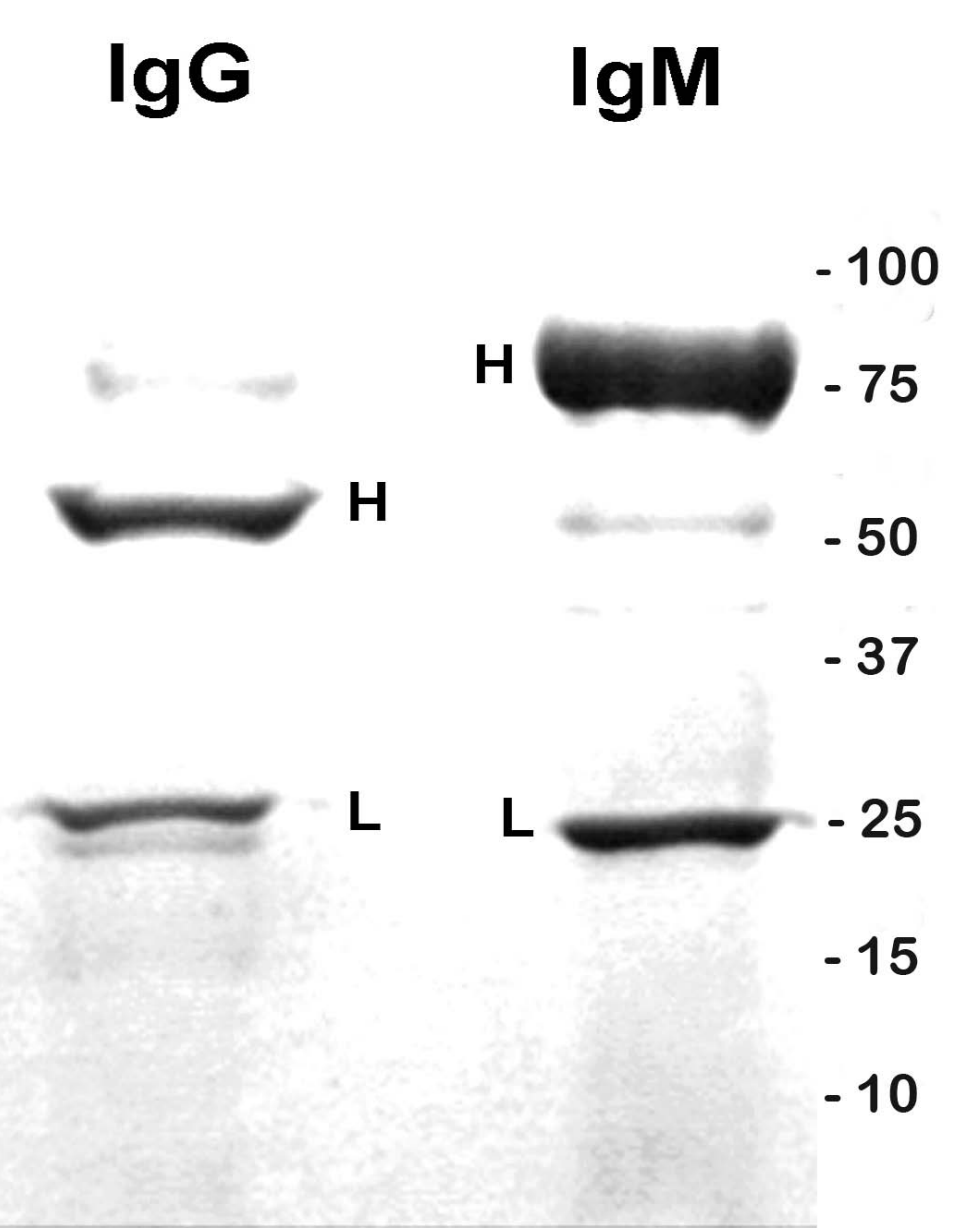
**SDS-PAGE of normal rat IgG and IgM used to assess immunoglobulin uptake by Purkinje cells**. SDS-PAGE demonstrates IgG bands at 55 kDa and 26 kDa MW and IgM bands of 75 kDa and 25 kDa. These molecular weights are consistent with IgG and IgM heavy and light chains and are labeled with "H" and "L" respectively. In non-reducing gels, both IgG and IgM produced single bands (data not shown). IgG or IgM breakdown fragments were not detected under either reducing or nonreducing conditions.

To preclude the possibility that immunolabeling for IgG or IgM might be due to potential IgG breakdown fragments, the normal rat IgG, the IgG monoclonal GK 1.5, and the normal rat IgM used in these experiments were analyzed by SDS-PAGE. Media containing each of the three immunoglobulins as used for initial culture incubation and after incubation at 37°C for 24 hours were analyzed under both under reducing (containing dithiothreitol) and non-reducing (lacking dithiothreitol) conditions. In reduced gels IgG produced bands at 55 kDa and 26 kDa MW, and IgM bands of 75 kDa and 25 kDa consistent with IgG and IgM heavy and light chains respectively (Figure 2). In non-reducing gels, both IgG and IgM produced single bands (data not shown). IgG or IgM breakdown fragments were not detected under either reducing or nonreducing conditions. No immunoreactivity with Purkinje cells was detected for the normal rat IgG, the anti-CD4 monoclonal, GK 1.5, or the normal rat IgM by immunofluorescence staining against fixed or unfixed frozen sections of rat cerebellum (See methods; data not shown), precluding the possibility that uptake of IgG by Purkinje cells in our cultures was the result of antibody binding to Purkinje cell membrane antigens.

### Uptake of IgG by Purkinje cells

**Figure 3:**
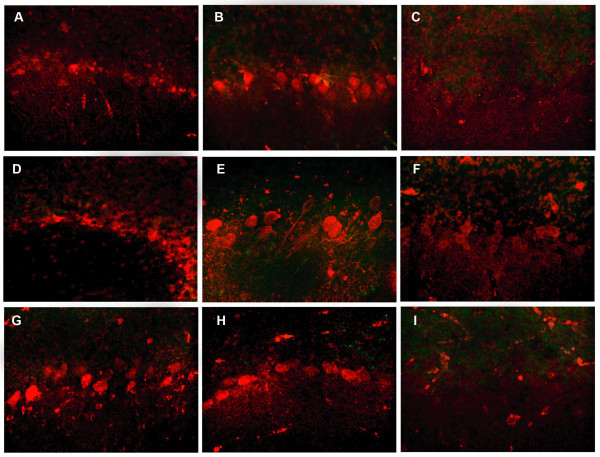
**Uptake and clearance of rat and human IgG and rat IgM by Purkinje cells over time**. Uptake of rat IgG (Panels A, B), human IgG (Panels D, E), and rat IgM (Panels G, H) (red) is demonstrated in Purkinje cells studied at 6 hours (Panels A, D, G), with more extensive uptake visible at 24 hours (Panels B, E, H). In these figures Cy5 labels immunoglobulins red. IgG or IgM are no longer detectable in cultures studied after 24-48 hour incubation after transfer to media lacking the immunoglobulin (Panels C, F, I). Yellow fluorescence, as would be indicative of SYTOX green staining of dead cells containing IgG was not apparent. (Objective magnification 40×)

Cultures incubated with normal rat IgG were harvested at time intervals from 2-48 hours. To confirm cell viability, cultures were reacted with SYTOX green for two hours prior to harvesting. Cultures were then fixed, mounted on glass slides and analyzed by confocal microscopy. The time course for uptake and clearance of the immunoglobulin is shown in Table 1. Bright immunofluorescence labeling specific for IgG was detected in Purkinje cell dendrites within 4 hours (Table 1) and in Purkinje cell bodies by 6 hours (Table 1)(Figure 3A). By 24 hours, IgG was clearly detectable in the majority of Purkinje cells throughout the cultures, with some cells showing IgG within cell nuclei as well as cytoplasm. (Figure 3B). Purkinje cells which incorporated IgG did not contain SYTOX green, indicating that these cells were viable at the time cultures were fixed. In other experiments, IgG-containing Purkinje cells in cultures maintained for 120 hours continued to exclude SYTOX green, indicating that the presence of IgG within Purkinje cells did not affect cell viability (data not shown). To confirm the identity of the cells taking up IgG as Purkinje cells, cultures were incubated with normal human IgG for intervals up to 96 hours, fixed, permeabilized, incubated with rabbit anti-calbindin antibody and then immunolabeled with Cy5-conjugated anti-human IgG and Cy3-conjugated donkey anti-rabbit IgG. Immunolabeling for IgG was shown to colocalize with immunolabeling for calbindin (Figure 1), confirming that the cells incorporating antibody were Purkinje cells. In these cultures, occasional IgG-containing cells were also detected which had the morphological characteristics of Purkinje cells but could not be shown to express calbindin (Figure 1); this is consistent with work by other investigators demonstrating that a minority of Purkinje cells in rat organotypic do not label with anti-calbindin antibodies [32]. Our results thus demonstrate that rat cerebellar cultures might potentially be suitable for studies of antibody-Purkinje cell interactions using human immunoglobulins.

**Table 1:** Time course of IgG and IgM uptake and clearance by Purkinje cells in organotypic cerebellar cultures

**Immunoglobulin**	**Immunoglobulin localization within Purkinje cells^a^**	**Immunoglobulin clearance from Purkinje cells^b^**
IgGs^c^	4 hours: Purkinje cell processes. Rare Purkinje cell bodies 6-8 hours: Purkinje cell processes and cytoplasm 24-48 hours: Most Purkinje cells (processes, cytoplasm, and nuclei)	24-48 hours

Normal Rat IgM	4 hours: Purkinje cell processes. Rare Purkinje cell bodies 6-8 hours: Purkinje cell processes and cytoplasm 24 hours: Most Purkinje cells (processes, cytoplasm, and some nuclei) 48 hours: Most Purkinje cells (processes, cytoplasm, and nuclei)	24-48 hours

a. Purkinje cell death was observed in <5% of cells through 120 hours of incubation as indicated by intracellular presence of the cell death marker, SYTOX green. Cell death was equivalent to that seen in cultures incubated without immunoglobulin.

b. Following transfer of cultures to media not containing the immunoglobulin

c. IgGs: normal rat IgG, GK1.5 monoclonal rat anti-CD4+ antibody, normal human IgG, Cy5-conjugated donkey anti-human IgG, FITC-conjugated donkey anti-human IgG. Cy5-conjugated donkey anti-human IgG and FITC-conjugated donkey anti-human IgG were studied both in real time in living cultures and following fixation.

We next incubated cerebellar cultures with human IgG to determine whether rat Purkinje cells were able to incorporate non-species immunoglobulin. Cultures were incubated with 7.5 μg/ml concentrations of normal human IgG, fixed, permeabilized, and labeled with Cy5-conjugated donkey anti-human IgG. Purkinje cell viability was confirmed by exclusion of SYTOX green. The time course of IgG uptake and intracellular distribution of human IgG was identical to that of rat IgG (Table 1). Human IgG was detectable in Purkinje cell processes within 4 hours, in Purkinje cell cytoplasm by 6 hours (Figure 3D), and in cell nuclei as well as cytoplasm by 24-48 hours (Figure 3E)(Table 1).

### Demonstration of IgG clearance from Purkinje cells

To determine whether Purkinje cells could clear IgG once it had been taken up intracellularly, organotypic cerebellar cultures were maintained in media containing rat IgG for 48 hours. The cultures were then transferred to media lacking rat IgG and studied after 24-72 hours. In these studies, Purkinje cells exhibited bright fluorescence specific for rat IgG after 48 hours of incubation with IgG. After transfer to IgG-free media, immunofluorescence was not detected after 24-48 hours, indicative of immunoglobulin clearance. (Figures 3C, 3F)(Table 1)

### Real-time confirmation of IgG uptake

**Figure 4:**
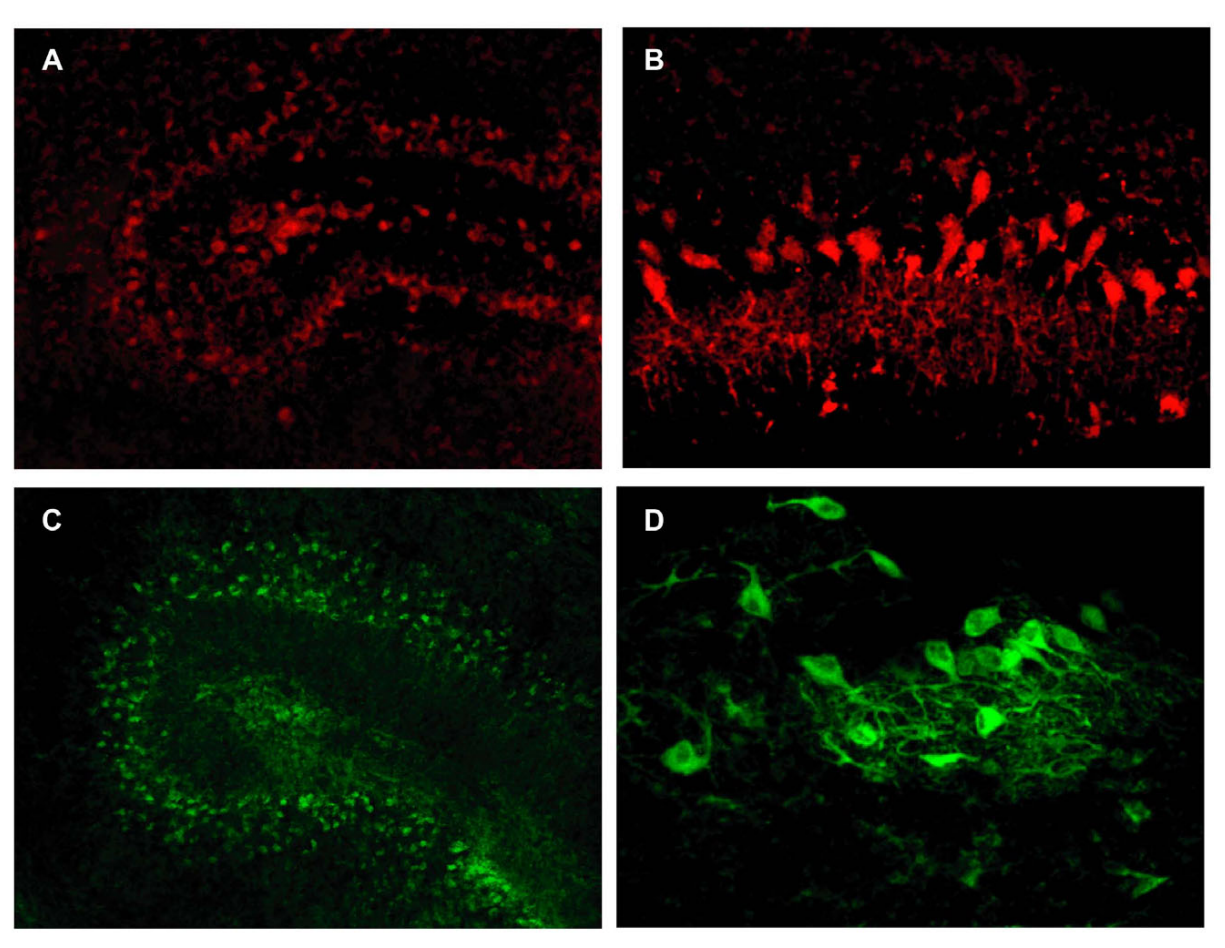
**Uptake in real time and in fixed tissues showing SYTOX exclusion and IgG clearance**. Cy5-labeled (red) and FITC-labeled (green) IgGs are demonstrated in Purkinje cell processes and cytoplasm in living cultures studied at 24 hours (Panels A, C)(Objective magnification 20×). Similar results were seen with tissues subsequently fixed and examined under higher magnification (Panels B, D)(Objective magnification 40×).

Organotypic cerebellar cultures were incubated with Cy5-conjugated or FITC-conjugated donkey anti-human IgG and studied over time in a heated microscope stage incubator (see methods). In these experiments, Cy5-labeled and FITC-labeled IgGs were detected in Purkinje cell dendrites and rare cell bodies within 4 hours (Table 1). By 24 hours, Cy5-conjugated (Figure 4A) or FITC-conjugated (Figure 4C) IgGs were present in the majority of Purkinje cells throughout the cultures. Similar results were seen with tissues subsequently fixed and examined under higher magnification (Figures 4B and 4D).

### Interaction of IgM with Purkinje cells

The ability of Purkinje cells to incorporate IgG raised questions as to whether these cells could also take up larger immunoglobulins such as IgM (molecular weight 950 kDa, compared to the 150 kDa weight of IgG). In cultures incubated with normal IgM, positive immunofluorescence staining for the immunoglobulin was detected within 4 hours in Purkinje cell processes (Table 1) and by 6-24 hours within the cytoplasm of most Purkinje cells, as well as within scattered Purkinje cell nuclei (Figures 3G, 3H). Viability of Purkinje cells incorporating IgM was confirmed by exclusion of SYTOX green. As with IgG, transfer of cultures to media lacking IgM resulted in disappearance of detectable immunofluorescence for the immunoglobulin within 24-48 hours (Figure 3I), indicative of clearance.

### Targeted killing of Purkinje cells by daunorubicin-conjugated IgG but not free daunorubicin

**Figure 5:**
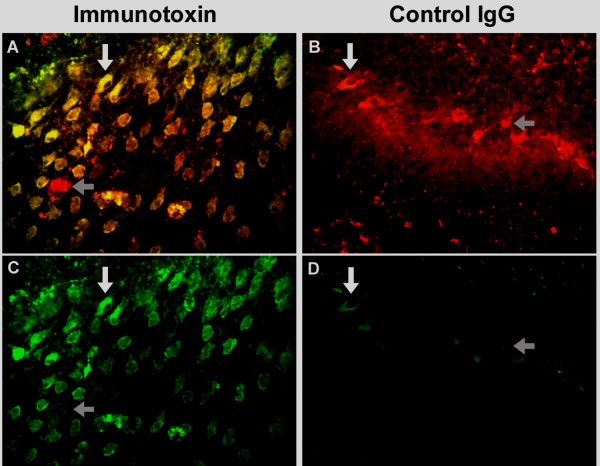
**Cultures incubated with GK1.5 IgG-daunorubicin immunotoxin or with GK1.5 antibody control, scored for cell death**. Cultures were incubated with either the immunotoxin (Panel A) or control IgG (Panel B) for 8 hours. SYTOX green was added during the last two hours of incubation to detect cell death prior to fixation and immunostaining for IgG. Cy5 labeling of IgG appears red. Intracellular presence of SYTOX, indicating cell death, appears green. Colabeling for IgG and SYTOX appears yellow. The SYTOX green channel is shown for the immunotoxin (Panel C) and control IgG (Panel D). Examples of cells scored as live cells (positive for IgG and negative for SYTOX green) are indicated with gray horizontal arrows, while examples of dead cells positive for IgG and SYTOX green are indicated with a white vertical arrow. (Objective magnification 40×)

**Figure 6:**
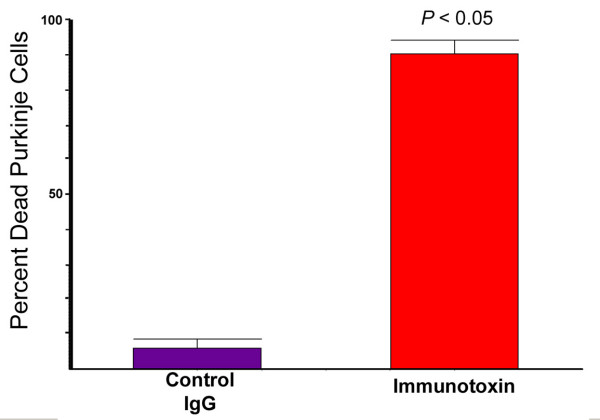
**Quantitation of Purkinje cell death in cultures incubated with IgG-daunorubicin immunotoxin for 8 hours**. Cell death was assessed from images such as those presented in Figure 5 (see methods). The average percent of cell death from four fields is shown for each treatment. Significance (*P *< 0.05) determined using the Mann-Whitney test.

Since Purkinje cells were shown to take up IgG, we investigated whether Purkinje cell-specific delivery of agents could be achieved by coupling them to IgG. To answer this question, we studied the ability of an IgG-immunotoxin to produce targeted Purkinje cell death. Organotypic cerebellar cultures were maintained in media containing 5 μg/ml of the daunorubicin-IgG immunotoxin and examined at 4, 6, 8, 24, and 48 hours. As controls, parallel cultures were incubated for equal periods of time with either the unconjugated IgG monoclonal or, to demonstrate that coupling to IgG was essential for daunorubicin uptake by Purkinje cells, with unconjugated daunorubicin present at a concentration (500 nM) equal to the total amount of daunorubicin contained in the IgG-immunotoxin conjugate (data not shown). SYTOX green was added to cultures during the final two hours before fixation. Presence of the IgG-daunorubicin complex within Purkinje cells and SYTOX green staining were selectively studied using confocal microscopy. The time course for uptake of each compound and their effect on cell viability are shown in Table 2. Within 4 hours after addition of the immunotoxin, uptake of the IgG-daunorubicin complex was observed within Purkinje cells as demonstrated by the intracellular presence of fluorescence specific for daunorubicin (data not shown) and by Cy5 immunolabeling specific for IgG. In cultures studied at 4 hours, scattered Purkinje cells contained SYTOX green, indicative of cell membrane injury and death. After 8 hours of incubation 90% of Purkinje cells containing the IgG-daunorubicin immunotoxin also contained SYTOX green indicative of cell death, as indicated by the presence of both IgG and SYTOX fluorochromes (Figures 5, 6). Uptake of IgG in control cultures paralleled that seen in cultures incubated with IgG-daunorubicin, but cell death was observed in less than 5% of cells (Figures 5, 6). Purkinje cells in organotypic cultures incubated with concentrations of free daunorubicin equal to those conjugated to the immunotoxin showed no evidence of uptake and no increase in numbers of dead cells (Table 2), indicating that the presence of IgG was required to achieve toxic intracellular concentrations of daunorubicin.

**Table 2:** Interaction of GK1.5 IgG monoclonal, free daunorubicin, and GK1.5-daunorubicin immunotoxin with Purkinje cells

**Reagent**	**Uptake by Purkinje cells**	**Purkinje cell death^a^**
GK1.5 monoclonal rat anti-CD4+ antibody	Identical to normal rat IgG (See Table 1)	<5% of cells^b^

Unconjugated (free) daunorubicin	No detectable uptake	<5% of cells^b^

GK1.5-daunorubicin immunotoxin	Initial uptake at 4 hours Widespread uptake by 6 hours	6 hours: <10% of cells 8 hours: 90% of cells

a. As indicated by intracellular presence of the cell death marker, SYTOX green in cells containing the IgG immunotoxin.

b. At 8 hours

## Discussion

Early in vivo studies suggesting potential uptake of IgG by cerebellar Purkinje cells were limited to methodologies employing fixed, post mortem material. These studies did not exclude the possibility of IgG entry into Purkinje cells after death, nor could they follow in real time, in a given animal, the course of IgG uptake, the effect of incorporated IgG on Purkinje cell viability, or whether these large neurons are able to clear IgG over time.

The present study demonstrates several important findings: 1) IgG is rapidly taken up by viable Purkinje cells in organotypic cultures as confirmed by colocalization of immunostaining for IgG and the Purkinje cell marker, calbindin. 2) The time course of IgG uptake by Purkinje cells in organotypic cultures parallels that described by Graus et al. in guinea pigs studied post mortem following intraventricular administration of IgG [14]. 3) IgG uptake can occur without specific antibody reactivity with Purkinje cell surface antigens. 4) Rat Purkinje cells are able to incorporate IgG from other species including humans, suggesting that this tissue culture system could potentially be used to study interaction of human antibodies with Purkinje cells. 5) Fluorochrome-conjugated (FITC or Cy5) IgG can be used to study antibody uptake by living Purkinje cells in real time. Intracellular IgG *per se *does not appear to affect Purkinje cell viability, and IgG is cleared from Purkinje cells after its removal from culture media. Somewhat surprisingly, viable Purkinje cells were also able to incorporate IgM, a much larger molecule. Our studies also demonstrated that Purkinje cells were also able to incorporate an IgG-daunorubicin immunotoxin complex and that the internalized immunotoxin rapidly caused Purkinje cell death without appreciable death of other neuronal populations over the time period studied. In contrast, Purkinje cells in cultures incubated with an equivalent amount of free daunorubicin remained viable, indicating that the uptake and subsequent toxic effect of daunorubicin for Purkinje cells required uptake of the toxin through its coupling to IgG. These data indicate that normal IgG can be used to target Purkinje cells for specific intracellular delivery of pharmacological agents. Although the present study dealt with a single cytotoxic agent, this method may potentially be applicable to Purkinje cell delivery of other IgG-conjugated molecules.

In our studies, both IgG and IgM appeared initially in Purkinje cell processes, followed by detection of IgG in cytoplasm and then in cell nuclei. These observations suggest that IgG within Purkinje cells accumulates in cell nuclei as well as cytoplasm. This finding has not previously been reported. However, intracellular trafficking of other proteins is well known to occur in neurons [33-36], and studies of molecules injected into non-neuronal cell types have demonstrated similar intracellular trafficking of proteins - including injected antibodies - from cytoplasm into cell nuclei [37-40].

IgM is a pentameric molecule with a molecular weight of 950 kDa, and given its large molecular weight, we had not expected it to be incorporated by Purkinje cells. Somewhat to our surprise, the molecule was readily incorporated into Purkinje cells and achieved intracellular cytoplasmic and nuclear distribution similar to that observed by IgG. As in the case of IgG, Purkinje cells appeared to tolerate intracellular IgM without effect on cell viability and were able to clear IgM following its removal from culture media.

The mechanisms by which Purkinje cells incorporate molecules are not well understood. Borges found that uptake of propidium iodide was blocked in vivo by pretreatment of animals with colchicine and also by ouabain, suggesting that uptake required active transport and possibly involved microtubules [9]. Whether similar mechanisms are involved in uptake of IgG or IgM is not known, and studies are currently in progress to determine whether uptake of IgG or IgM can be blocked using these compounds or other agents known to interfere with active transport across cell membranes.

The ability of cerebellar Purkinje cells to incorporate not only IgG but also IgG-conjugated immunotoxins may have applicability to the use of monoclonal IgGs or IgG immunotoxins in the treatment of human disease. Two early animal studies involving intraventricular injection of immunotoxins demonstrated Purkinje cell loss. Muraszko et al. noted Purkinje cell loss in rats and monkeys receiving intrathecal injections of ricin A chain conjugated to monoclonal antibodies reactive with transferrin receptors [18]. These authors attributed Purkinje cell loss to immunotoxin-induced CSF eosinophilia [18]. Davis and Wiley demonstrated Purkinje cell loss in rats after intraventricular injection of saponin conjugated to OX7, an antibody directed against Thy1 [19]. The antibodies used by both Muraszko et al. and by Davis and Wiley were known to react with antigens present on Purkinje cells [41-43]. In contrast, Pai et al. treated 23 patients with advanced, refractory epithelial carcinoma of the ovary with intraperitoneal infusions of pseudomonas exotoxin conjugated to a mouse monoclonal IgG_2b _antibody, OVB3 (OVB3-PE). OVB3 had been generated by immunizing mice with cell membranes from the human ovarian carcinoma cell line, OVCAR-3 [20]. OVB3-PE had been found to be safe in studies of mice and monkeys and to be effective against all ovarian cancers tested in culture [20,44-46]. The study was terminated, however, after three patients developed severe, in one case fatal encephalopathies. In the patient developing fatal disease, CSF was found to have extreme elevation of protein. MRI showed areas of increased signal and gadolinium enhancement in cerebellum, deep cerebellar nuclei, and brainstem. Animal studies with OVB-PE had not demonstrated reactivity with neural tissue. However, our study suggests that uptake of the immunotoxin into Purkinje cells and possibly other neurons could have occurred without actual antibody specificity. The ability of Purkinje cells to incorporate IgG and IgM may have relevance to the role of antibodies in paraneoplastic or other neuronal death. In addition, the ability of Purkinje cells to incorporate molecules conjugated to IgG provides a potential tool for pharmacological studies involving Purkinje cells and also a cautionary note for the use of IgG conjugates in treatment of human disease.

## Conclusion

We conclude that Purkinje cells in rat organotypic cultures are able to take up and clear host (rat) and non-host (donkey or human) IgG independent of the immunoglobulin's reactivity with Purkinje cell antigens. The cells are also able to incorporate and clear IgM. This property permits specific targeting of Purkinje cells using both fluorochrome IgG conjugates, allowing study of Purkinje cell-IgG interaction in real time, and also IgG immunotoxins or possibly conjugates of other pharmacological agents. Our studies suggest that paraneoplastic or other autoantibodies reactive with nuclear or cytoplasmic Purkinje cells antigens might also be taken up intracellularly and could potentially produce cell injury and death. Our data also indicate that antibodies used therapeutically, including monoclonal agents and immunotoxins could also be taken up and cause Purkinje cell injury, even if they do not recognize Purkinje cell antigens.

## Competing interests

The authors declare that they have no competing interests.

## Authors' contributions

KEH and JEG planned the study, interpreted the data, and prepared the manuscript. KEH purified the monoclonal antibodies used in the study, performed the studies involving daunorubicin, and carried out the confocal microscopy and photomicrography. SAC assisted in planning the tissue culture studies and was responsible for tissue culture work and gel analysis of immunoglobulins. JWR developed the immunotoxin reagents employed in the study and assisted in preparation of the manuscript. NGC assisted in planning the research, interpreting the data, and preparing the manuscript.
